# Intraoperative hypothermia in patients undergoing Total knee arthroplasty: a cross-sectional study from a developing country

**DOI:** 10.1186/s12891-021-04390-7

**Published:** 2021-05-31

**Authors:** Ronika Devi Ukrani, Aiman Arif, Anum Sadruddin, Obada Hasan, Shahryar Noordin

**Affiliations:** 1 Medical College, Aga Khan University Hospital, Karachi, 74800 Pakistan; 2 Department of Orthopaedic Surgery, Aga Khan University Hospital, Karachi, 74800 Pakistan

**Keywords:** Developing country, Hypothermia, Intraoperative hypothermia, SSI, Total knee arthroplasty

## Abstract

**Background:**

Intraoperative hypothermia is associated with various risk factors, morbidity, and mortality in patients undergoing total knee arthroplasty (TKA), increasing the emotional and financial burden on patients. This study aimed to identify risk factors of intraoperative hypothermia in patients undergoing TKA.

**Materials and methods:**

All adult patients (⩾18 years) who underwent TKA from January 2016 to December 2017 at a tertiary-care hospital in Pakistan were included in this retrospective, cross-sectional study. Temperature < 36 °C was defined as hypothermia.

**Results:**

The study included 286 patients (77.6% female) with a mean age of 61.4 ± 10.4 years. The overall proportion of intraoperative hypothermia was 26.6%. Of the total patients, 66.1% underwent bilateral TKA whereas 33.9% underwent unilateral TKA. 73.8% of the patients were ASA Level 2. Only 13.3% of patients had postoperative hypothermia.

**Conclusion:**

Intraoperative hypothermia was significantly associated with age, bilateral procedure, ASA level and postoperative hypothermia in patients undergoing TKA. The surgeon and the operative team should be aware of the risk factors and the adverse outcomes associated with intraoperative hypothermia, especially in resource constrained settings to plan preventive strategies.

**Trial registration:**

This study was retrospectively registered on ClinicalTrials.gov on 3rd October 2020. The registration ID is NCT04575246.

## Introduction

Hypothermia, defined as core body temperature < 36 °C [1, 2], is a phenomenon in which the rate of heat loss is greater than the rate of heat production. It results from increased heat redistribution from core to periphery, coupled with decreased metabolic heat production [3].

Intraoperative hypothermia is a common complication of anaesthesia administration that affects normal thermal autoregulation in the body [4, 5]. Moreover, further heat loss due to the body surface area exposed to the cold operating room environment [6, 7] means that normal metabolic heat production is inadequate to maintain the core body temperature of the patient leading to numerous avoidable complications by altering normal physiologic processes in the body. The result of drastic heat loss is systemic vasoconstriction and reduction in peripheral circulation which leads to several different events such as tissue hypoxia [8], failure of immune cells to reach target cells and altered neutrophil function which are a major cause of post-operative infection [6, 8, 9]. Other complications include platelet dysfunction leading to bleeding tendencies and increased requirement for blood transfusions [3, 8, 9], and cardiac events such as tachycardia due to increased catecholamine levels causing a detrimental impact on morbidity and mortality of patients [6, 8, 9]. Post-operative shivering can also lead to unnecessary discomfort and prolonged hospitalizations for the patients [6, 8, 9].

The occurrence of perioperative hypothermia in total joint arthroplasty has been assessed in several studies and has yielded a range of results ranging from 11 to 72% [4, 10–12] to even up to 90% [7]. A study reported that 34% of patients undergoing total knee arthroplasty (TKA) have hypothermia at the time of incision and 33% of the patients stayed hypothermic throughout surgery [7]. Moreover, in the absence of active warming techniques, a 1–2 °C fall in temperature has been observed in surgical procedures [3].

The increase in the incidence of untoward complications as a result of hypothermia are a cause of concern for patients and surgeons. TKA is a procedure frequently done in elderly patients with end-stage osteoarthritis at our University Hospital. Although previous literature on TKA and hypothermia exists from developed countries, there is a scarcity of data from developing countries for complications hypothermia. The differences in settings, resources and risk factors in developing countries merit further research to enhance the clinical course of these patients. The purpose of this study is to determine the frequency of intraoperative hypothermia and factors associated with intraoperative hypothermia in patients undergoing TKA at a tertiary care hospital in Karachi, Pakistan. This can allow surgical teams in developing countries in the future to determine the risk factors that can be controlled to improve postoperative clinical course with regards to intraoperative hypothermia.

## Materials and methods

### Study location and sample

We conducted a retrospective cross-sectional study of patients who underwent total knee arthroplasty at the Aga Khan University Hospital (AKUH) between the period January 2016 to December 2017. Nonprobability consecutive sampling was used for all patients fulfilling the inclusion criteria which was adults ⩾18 years undergoing TKA at our University Hospital.

The study was conducted in accordance with the ethical standards described in the 1964 Declaration of Helsinki and its later amendments. Aga Khan University Ethics Review Committee (ERC) approval was obtained prior to the initiation of this study. The ERC study protocol number is 2019–1274-3270. The study was approved with an exemption of informed consent from patients due to lack of contact with them in a retrospective clinical study. Data was collected from patient charts and files without any interaction with the patient.

### Patients and methods

A standardized proforma was used to collect data from an online patient data system about patients’ preoperative, intraoperative, and postoperative details. Preoperative data included age, gender, and comorbid conditions. Intraoperative data was collected from the surgical and anaesthesia records and included surgical site, American Society of Anesthesiologists (ASA) Level, duration of surgery, preoperative body temperature, intraoperative body temperature and duration of surgery. Postoperative data included postoperative body temperature and the presence of any surgical site infection within the 1 year follow-up period.

### Standard operating conditions

All TKA procedures at AKUH are conducted under a standard protocol. The operating room temperature is set at 20 °C. Moreover, all patients are given a bear hugger with K-thermia underneath set at 37 °C.

### Statistical analysis

IBM SPSS (Statistical Package for Social Sciences) Version 23.0 was used for all statistical analysis. Continuous data is presented as mean ± standard deviation with comparison using independent sample t-test. Nominal and ordinal data is presented as N (%) with percentages compared using Chi-Square test. A *p*-value of < 0.05 was considered significant for all analyses.

## Results

The study comprised of a total of 286 patients with 77.6% of the patients being female. The mean age of the patients was 61.4 ± 10.4 years. 64.3% of the patients were hypertensive and 27.6% were diabetic. The indication for performing TKA was predominantly osteoarthritis. Of the 286 patients, 189 underwent bilateral TKA, while 97 patients underwent unilateral TKA (right: 46; left: 51). The majority of the patients were classified as ASA Level 2 (73.8%) followed by ASA Level 3 (20.3%) and ASA Level 1 (5.9%). The mean duration of surgery was 201.2 ± 66.2 min. The mean duration of surgery in patients who underwent unilateral TKA was 131.4 ± 36.1 min as opposed to 237.0 ± 46.6 min in patients who had bilateral TKA (*p* = 0.032). Preoperatively, 62 (21.7%) patients had hypothermia. However, 76 (26.6%) patients developed intraoperative hypothermia while 38 (13.3%) patients were noted to be hypothermic postoperatively.

The mean age of patients in the intraoperative hypothermia group was 63.7 ± 10.1 years which was significantly higher than the mean age of patients in the normothermia group (60.5 ± 10.5 years; *p* = 0.023). In the intraoperative hypothermia group, 53.9% of patients underwent bilateral TKA (*p* = 0.018) which was noted to be statistically significant. There were more patients classified as ASA Level 2 in the intraoperative group than in the normothermia group (84.2% vs. 70.0%; *p* = 0.049). A significantly lower proportion of intraoperative hypothermia patients had hypothermia postoperatively (23.7% with *p* = 0.002). Only 2 patients developed surgical site infection postoperatively.

The results of the study are shown in Table 1.

**Table 1:** Characteristics of TKA Patients

Variables	Overall (***N*** = 286)	Intraoperative Hypothermia		***p***-value
		Yes (***N*** = 76)	No (***N*** = 210)	
**Age**	61.4 ± 10.4	63.7 ± 10.1	60.5 ± 10.5	**0.023**
**Gender**				0.200
Male	64 (22.4)	21 (27.6)	43 (20.5)	
Female	222 (77.6)	55 (72.4)	167 (79.5)	
**HTN**				0.418
Yes	184 (64.3)	46 (60.5)	138 (65.7)	
No	102 (35.7)	30 (39.5)	72 (34.3)	
**DM**				0.551
Yes	79 (27.6)	19 (25.0)	60 (28.6)	
No	207 (72.4)	57 (75.0)	150 (71.4)	
**Rheumatoid Arthritis**				
Yes	2 (0.7)	0 (0)	2 (1.0)	> 0.999
No	284 (99.3)	76 (100)	208 (99.0)	
**Osteoarthritis**				0.252
Yes	9 (3.1)	4 (5.3)	5 (2.4)	
No	277 (96.9)	72 (94.7)	205 (97.6)	
**Site**				**0.018**
Right	46 (16.1)	19 (25.0)	27 (12.9)	
Left	51 (17.8)	16 (21.1)	35 (16.7)	
Bilateral	189 (66.1)	41 (53.9)	148 (70.5)	
**ASA Level**				**0.049**
Level 1	17 (5.9)	2 (2.6)	15 (7.1)	
Level 2	211 (73.8)	64 (84.2)	147 (70.0)	
Level 3	58 (20.3)	10 (13.2)	48 (22.9)	
**Preoperative Hypothermia**				0.620
Yes	62 (21.7)	18 (23.7)	44 (21.1)	
No	224 (78.3)	58 (76.3)	166 (79.0)	
**Postoperative Hypothermia**				**0.002**
Yes	38 (13.3)	18 (23.7)	20 (9.5)	
No	248 (86.7)	58 (76.3)	190 (90.5)	
**SSI**				0.462
Yes	2 (0.7)	1 (1.3)	1 (0.5)	
No	284 (99.3)	75 (98.7)	209 (99.5)	
**Duration of Surgery (mins)**	201.2 ± 66.2			
Unilateral	131.4 ± 36.1	126.4 ± 38.6	135.2 ± 33.8	0.2466
Bilateral	237.0 ± 46.6	228.3 ± 49.2	240.4 ± 45.3	0.1246

## Discussion

The incidence of intraoperative hypothermia in patients undergoing TKA is increasing and is expected to rise to 600% by 2030 [13]. We conducted a retrospective single centre study to determine risk factors of intraoperative hypothermia in patients undergoing TKA. The results of our study highlight that age, bilateral procedure, ASA level and postoperative hypothermia were significantly associated with intraoperative hypothermia.

The incidence of intraoperative hypothermia reported in our study was 26.6%. The findings of our study are similar to the results of a study conducted by Frisch et al. who reported 32.6% patients with intraoperative hypothermia during TKA [13].

The mean age of patients in our study was 61.4 ± 10.4 years, with intraoperative hypothermia group having a significantly higher age than normothermia group (*p* < 0.023). A previously reported study also had mean age of patients 62 ± 13 years which is similar to the finding of our study [14]. Another study conducted in United Kingdom had higher age in hypothermia group compared to normothermia group [2]. Yinan Li et al. also conducted a retrospective study including 1467 patients who underwent video assisted thoracoscopic surgery and reported an increased risk of intraoperative hypothermia with advanced age [15]. Age increases the risk of osteoarthritis and as a result, patients get TKA for management. This association between intraoperative hypothermia and older patients could also be due to decrease in thermoregulatory vasoconstriction threshold in patients under general anaesthesia with age [15].

The percentage of female patients who have had TKA reported in literature vary between approximately 56–74%. Although, the reason for higher prevalence of TKA among females is unclear, the greater percentage of females in our cohort (77.6%) could be attributed to women being more prone to injuries [12, 13, 16, 17]. Female gender is also considered the strongest risk factor for knee osteoarthritis [17].. In addition, female gender is also a risk factor for hypothermia due to smaller reserve of body fat [7, 18]. The findings of our study show that among the patients in the intraoperative hypothermia group, majority of the patients were female (72.4%) and only 27.6% were male. This is also evidenced by the high percentage of 79% females in the study at AKU Hospital by Usmani et al. [19] The patients in our study had multiple co-morbidities, with hypertension (64.3%) being the most common. The prevalence of hypertension in our study is similar to the prevalence of hypertension previously reported in patients undergoing TKA [20].

Majority of the patients in our study had bilateral TKA (66.1%) and a significantly greater number of patients in intraoperative hypothermia group had bilateral TKA (53.9%; *p*-value = 0.018). Although our findings show that bilateral TKA increased the risk of intraoperative hypothermia compared to a unilateral procedure, there is lack of data showing the correlation of bilateral procedures and intraoperative hypothermia in patients for total knee arthroplasty. Theoretically, in bilateral procedure, the surgical site is exposed to lower temperatures for a total longer duration during sequential surgery, hence there is an increased risk of hypothermia. A study conducted by Qadir et al. reported a higher incidence of sequential bilateral TKA compared to staged TKA in Pakistan [21]. However, more research is required to assess the effect of bilateral TKA on hypothermia.

According to the results of our study, the most common ASA Level was Level 2. In a study conducted by Vural et al. inadvertent hypothermia, in patients from several different fields including orthopedic surgery, was most commonly associated with ASA Level 2 which is consistent with our findings [22]. This study also reported that ASA level had an impact on intraoperative body temperature and was a risk factor of development of hypothermia in univariate analysis [22]. However, in multivariate analysis, ASA Level had no effect on hypothermia [22]. Moreover, studies conducted by Belayneh et al. [23] and Monzón et al. [24] also reported that ASA Level was a risk factor for development of hypothermia. The results of our study reported that ASA Level was significantly associated with intraoperative hypothermia (*p* = 0.049). However, more studies are still required to establish an association between ASA classification and development of intraoperative hypothermia in a developing country.

According to Vural et al., intraoperative hypothermia is a risk factor for postoperative hypothermia [22]. Although our results showed that postoperative hypothermia is significantly associated with intraoperative hypothermia (*p* = 0.002), the incidence of postoperative hypothermia in intraoperative hypothermia group was lower than in normothermia group. The difference in the results can be due to differences in postoperative care.

Only 2 (0.7%) patients developed SSI post-operatively with only 1 (1.3%) out of total 76 hypothermic patients having suffered infection which was non-significant. Thereby, our study could not find a positive association between intraoperative hypothermia with wound infection, as suggested by other studies [13].

Further studies are required in order to better understand the risk factors associated with hypothermia and its impact on different post-operative outcomes. In addition, further studies can also help devise standardized protocols such as effective warming techniques as preventative measures to reduce hypothermia and to reduce the risk of complications that may arise due to it.

### Caveats

There were several limitations in this study. The sample size was small which could be one of the reasons the post-operative hypothermia results were inconsistent with the literature. Body surface area and BMI was not included in the study. Measurement of temperature was not standardized and data on duration of hypothermia and rewarming strategies was also not recorded. ASA Level 4 category patients are not candidates for quality of life surgery and hence were not included which potentially skews the understanding of the association between ASA and hypothermia as the entire spectrum is not included.

## Conclusion

Intraoperative hypothermia is significantly associated with age, bilateral procedures, ASA level and postoperative hypothermia in patients undergoing TKA. The surgeon and the operative team should be aware of the risk factors and the adverse outcomes associated with intraoperative hypothermia, especially in a resource constrained developing country like Pakistan.

## Data Availability

The datasets generated and/or analysed during the current study are not publicly available since the Ethical Review Committee guidelines do not allow institutional data to be dispersed. However, the data is available on reasonable request to the corresponding author.

## Abbreviations

TKA: Total Knee Arthroplasty
AKUH: Aga Khan University Hospital
ASA Level: American Society of Anesthesiologists (ASA) Level
SSI: Surgical Site Infection
BMI: Body Mass Index
HTN: Hypertension
DM: Diabetes Mellitus
